# Experiences of Women Faculty in an Academic Medical Center

**DOI:** 10.1089/whr.2024.0138

**Published:** 2025-03-13

**Authors:** Charlotte R. Esplin, Lisa Calderwood, Chantel M. Weisenmuller, Jessica L. Luzier

**Affiliations:** ^1^ Charleston Area Medical Center, Charleston, West Virginia, USA.; ^2^ School of Medicine – Charleston Division, West Virginia University, Charleston, West Virginia, USA.; ^3^ Sam Houston State University, Huntsville, Texas, USA.

**Keywords:** academic medical centers, gender equity, burnout, psychological, women, job satisfaction, parenting

## Abstract

**Background:**

Women faculty face different obstacles in academic medical careers than their men counterparts. Women faculty report feeling like “outsiders” and experiencing multiple barriers to career advancement compared with men, especially if they hold a nonmedical degree (*e.g.*, PhD). This study examined aspects of workplace culture that differentially impact women at a large regional academic medical center (AMC) in the Appalachian region of the United States—a geographic area that is largely understudied in this body of literature.

**Materials and Methods:**

Forty-seven women completed a survey that included the Culture Conducive to Women’s Academic Success instrument, the Professional Fulfillment Index, the Work and Family Conflict scale, and items measuring burnout, childcare availability, and demographic factors.

**Results:**

Our findings revealed that many women faculty felt that they were being treated differently than men faculty, that work infringed on their home and family life, and that while they were professionally fulfilled at work, childcare problems exacerbated feelings of wanting to leave that AMC. About 60% of our sample indicated some level of burnout.

**Conclusions:**

These findings align with previous findings that women juggle multiple roles that are typically not expected of men, and this juggling may be one reason why women are not staying in academic medicine or being promoted at the same rate as men. We provide incremental validity for the measures used and delineate specific ideas for improvement, such as on-site childcare, standardized leave policies, and formal mentorship and curriculum programs.

## Introduction 

Careers in academic medical centers (AMCs) are heralded as opportunities to blend both clinical and research productivity.^1^ Yet, decades of scientific research have demonstrated that women working in AMCs may have experiences that are less favorable than those of their colleagues who are men.^2^ Women faculty report feeling like “outsiders,” especially if they are a woman of color or hold a nonmedical degree, and experiencing multiple barriers to career advancement.^3^ Nationwide, women faculty are not advancing in their careers at the same rate or frequency as men,^4–6^ are likely to earn less than men,^4,7,8^ receive no or poor mentorship,^9,10^ are given fewer hard and soft resources (*i.e.*, lab space and research funding),^11,12^ and are less likely to reach leadership positions compared with men.^5,6,13^ In addition, women faculty tend to engage in more uncompensated internal service,^2^ which is factored into annual evaluations but generally carries less weight than other criteria in the promotion and tenure process. Overall, the work lives of women faculty are vastly different from their men counterparts. A report issued by the National Academies, *Beyond Biases and Barriers,* explained, “the problem is not old-style, overt sex discrimination, but rather unrecognized features of the organizational culture that affect men and women differently.”^14^

If a woman remains in academic medicine long enough to be considered for promotion, she faces more difficulty in career advancement than a man would.^15^ For example, women make up more than 50% of graduating medical students but are only 10% of academic department chairs and 17% of tenured professors.^13,16^ These discrepancies indicate that somewhere during their early career, women are not being promoted at the same rates as men, which results in decreased representation of women at the top levels of academic medicine.^15^ Clearly, the numbers show that women have not yet reached equality when it comes to representation at higher levels of career advancement.

Even when women are promoted to leadership positions, they report having different experiences than the men who are their colleagues. Women faculty in leadership experience more social ostracism and “pushback” from their colleagues when they implement changes as part of their leadership responsibilities.^17^ Furthermore, women leaders are expected to perform more departmental “housekeeping” tasks and volunteer their time more often than their men counterparts.^9^ While time-consuming, these service tasks are not as highly valued in the promotion and tenure process. Furthermore, having fewer women faculty of advanced academic ranks results in fewer women mentors and role models.^9^ This may negatively impact role socialization, sense of belonging in the workplace, and the availability of peer networks for collaboration for junior women faculty.^18^

Extant research has demonstrated that physicians are more burned out than the general population.^19,20^ Notably, burnout disproportionately affects women physicians.^21,22^ In a study of family physicians and general internists, Rabatin and colleagues reported that women physicians were almost twice as likely as men to report burnout (36% vs. 19%, *p* < 0.001).^23^ In a review of over 40 studies published between 2010 and 2019, nearly 90% of the studies demonstrated higher rates of burnout in women physicians than in their men counterparts, especially in the dimension of emotional exhaustion.^24^ In addition to feeling burned out, women physicians also report lower levels of professional fulfillment in their jobs, which has been tied to leaving a career in medicine entirely.^21^ Burnout could pose a reasonable explanation as to why some women faculty are leaving their careers, resulting in fewer women faculty of advanced rank in academic medicine.

Parenthood has been cited as the most common barrier women face to taking a leadership role in medicine.^25–27^ Women who have children are impacted by the lack of standardized parental leave policies, conflicting demands from work and home life, and the availability of childcare.^28,29^ At one AMC, women faculty cited lack of paid maternity leave as a barrier to career advancement and a barrier to retention of women faculty.^9^ Being forced to use up their sick days and vacation days, in the absence of parental leave, meant that women faculty had no time to take when they inevitably experienced illness and contributed to burnout due to having no time off for recreation.^9^ Finding reliable and safe childcare options is also difficult for most women faculty. Health care workers frequently work far outside of the typical operating hours of most childcare providers. Arranging work schedules to accommodate childcare responsibilities is very stressful and can negatively affect career advancement.^28,29^ Harry and colleagues surveyed physicians and nurses and discovered that childcare stress was higher among women and people of color. Furthermore, respondents with childcare stress were 80% more likely to be burned out, and 28% more likely to intend to leave their jobs.^30^ For these and other reasons, many women feel that they must choose between advancing in their medical career or creating a family.

Given that women faculty have different experiences when compared with men in academic medicine, the current study examined aspects of workplace culture that differentially impacted women at a large regional AMC in the Appalachian region of the United States—a geographic area where gender differences in academic medicine have been largely understudied. We also propose future directions to overcome the identified challenges.

## Methods

### Procedures

The AMC’s Institutional Review Board (IRB) approved this study IRB Number 22-852 as Expedited Review Category 7, deeming it to have Not Greater than Minimal Risk under 45 CFR 46/21 CFR 56. This study utilized a prospective, comprehensive assessment given to credentialed faculty (*e.g.,* MD, DO, PhD *etc.*) who identified as women at an AMC in the Appalachian region of the United States. Participants were recruited *via* email using lists of credentialed medical faculty (including both men and women) who were on staff at this AMC. The email invited women to participate in the assessment survey by following a link. Three follow-up reminders were sent. Inclusion criteria included 18 years of age or older; credentialed medical staff member; and identification as a cisgender woman, transgender woman, or gender-diverse person.

### Measures

We compiled a comprehensive assessment by employing several validated measures (detailed below) and supplementing them with questions that we developed as needed to understand the challenges encountered by women faculty at our AMC.

#### Demographic questions

Participants were asked to self-report demographic and identity characteristics, including age, race, ethnicity, and gender identity.

#### Questions related to family care

Questions included those about academic rank, family makeup, family leave, and local childcare. These questions were developed based on focus groups conducted by the Women in Medicine group at the AMC and a review of pertinent factors related to women’s academic medicine careers identified through a literature search.

#### Work–family conflict

The Multidimensional Measure of Work Family Conflict (MMWFC) measure was used to evaluate two constructs: time-based work–family conflict and strain-based work–family conflict.^31^ Time-based work–family conflict describes when the time demands of work get in the way of effective participation in a family role or the time demands of family life affect work. Strain-based work–family conflict describes when strain/stress from work negatively affects family life or strain/stress from family life affects work. The MMWFC uses three items to assess each of these scales, rated on a 5-point Likert scale from 1 (strongly disagree) to 5 (strongly agree). The research team added two final questions to capture work-based interference with self-care, as well. Higher scores on this measure indicate higher levels of conflict between one’s family life and one’s work life.

#### Culture Conducive to Women’s Academic Success

The Culture Conducive to Women’s Academic Success (CCWAS) assessment instrument was developed through a combination of literature reviews, focus groups, and consultation with subject matter experts and aims to assess the supportiveness of the culture for women in academic medicine.^32^ The CCWAS consists of four dimensions: equal access, work–life balance, freedom from gender biases, and supportive leadership. The instrument has 45 questions rated on a 5-point Likert scale from “strongly agree” to “strongly disagree.” The “culture” as defined in this instrument is assessed at the department/unit level. Higher scores on this measure indicate a more supportive environment for women.

#### Screener for burnout

Dolan and colleagues developed and tested a single-item screener for burnout in health care workers.^33^ This item reads, “Overall, based on your definition of burnout, how would you rate your level of burnout?” Scores ≥3 are indicative of 1 or more symptoms of burnout.

#### Measure of physician well-being and burnout

The Professional Fulfillment Index (PFI) is a short, self-report measure to assess faculty levels of burnout and professional fulfillment.^34^ It captures information on 16 items, rating the participant’s experiences over the past 2 weeks. Component analysis revealed three subscales: professional fulfillment, work exhaustion, and interpersonal disengagement. The average item cutoff score across scales for burnout was 1.33 or higher (scale ranged from 0 to 4).

### Data analysis

We used STATA version 18 for all data cleaning and statistical analyses (StataCorp., 2023). Missingness was calculated to be 11%, so we created a missingness variable and used it to explore whether it was correlated with our independent and dependent variables. There were no significant correlations with either independent or dependent variables, so we deemed our missingness to be missing completely at random and proceeded with analyses. We ran scatter plots of each of our measures to check for outliers, but no outliers were found. We reverse-coded negatively worded items on the CCWAS (*i.e.*, items 10, 14, 21, 22, 25, 26, 27, 28, and 32) so that the use of a total scale score would make mathematical sense. However, as we only report the subscale scores for the PFI, we did not recode negatively worded items on this scale (*i.e.*, items 7–16).

We used G*Power to determine that given our small sample size, this study was underpowered to detect small effects when using inferential statistics (*e.g.*, analysis of variance, regression, and t tests). As a result, we used basic descriptive statistics to understand the responses from our sample.

## Results

### Study sample

Of the 600 credentialed faculty providers at the AMC who were emailed for survey participation, 83 responded for an initial response rate of 13.8%. Surveys were then excluded for the following reasons: did not answer “yes” to informed consent (*N* = 10), reported male gender (*N* = 23), did not answer the gender item (*N* = 2), or was a duplicate submission (*N* = 1). After these exclusions, 47 surveys were included in the analyses for a final estimated response rate of 7.8%. All were completed by cisgender women. Most had no academic rank or were in private practice (24 faculty participants, 51.1%), 11 were assistant professors (23.4%), 10 were associate professors (21.3%), 2 were full professors (4.3%), and 1 declined to answer (2.1%). Faculty with no academic rank were typically clinical faculty who regularly trained medical learners. Compared with national data from the American Association of Medical Colleges (AAMC), some academic ranks were likely underrepresented among the survey respondents (*e.g.*, nationally 28% of full professors are women,^35^ but only 4% in our sample). In our sample, 85% of women participants reported being currently partnered (*N* = 40), whereas 15% reported being single (*N* = 7). Regarding parenthood, 76% of our sample (*N* = 36) reported having one or more children (range 0–4 children), and the average number of children per participant was 1.5 (standard deviation [SD] 1.2). The average age of participants’ youngest child was 10 (SD 9.3). Table 1 provides further descriptive information regarding our sample.

**Table 1.** Participant Demographics

Characteristic	*N* (%)^a^
Study population	
Cisgender women	47 (100%)
Race	
White	37 (78.7%)
Black/African American	3 (6.4%)
Asian	3 (6.4%)
Native Hawaiian or Pacific Islander	3 (6.4%)
American Indian or Alaska Native	1 (2.1%)
Ethnicity	
Not Hispanic or Latino	46 (97.9%)
Hispanic/Latinx	1 (2.1%)
Parenthood	
One or more children	36 (76.6%)
No children	11 (23.4%)
Number of children, mean ±SD	1.5 ± 1.2
Age of youngest child, mean ±SD	10 ± 9.3
Specialty	
Behavioral medicine/psychiatry	7 (14.9%)
Pediatrics	7 (14.9%)
Family medicine	6 (12.8%)
Anesthesia	5 (10.6%)
General, vascular, and craniomaxillofacial surgery	3 (6.4%)
Cardiology	2 (4.3%)
Emergency medicine	2 (4.3%)
Obstetrics/gynecology	1 (2.1%)
Internal medicine	1 (2.1%)
Internal medicine/psychiatry	1 (2.1%)
Neurology	1 (2.1%)
Hematology/oncology	1 (2.1%)
Pulmonary	1 (2.1%)
Other ^b^	9 (19.2%)
Academic rank	
No academic rank	21 (44.7%)
Assistant professor	11 (23.4%)
Associate professor	10 (21.2%)
Full professor	2 (4.3%)
Private practice	3 (6.4%)
Preferred not to answer	1 (2.1%)

^a^ Unless otherwise specified.

^b^ “Other” specialties included endoscopy, orthopedics, and gastroenterology.

SD, standard deviation.

### Work environment for women faculty

To investigate the working environment for women faculty in their departments and in the hospital in general, we examined scores from the CCWAS. Higher scores indicate a more supportive perceived working environment for women. Respondents identified room for improvement in all four of the dimensions queried (Table 2). Nearly half of our respondents reported perceptions that women were not considered for leadership positions as frequently as men (42%), perceptions that their chair/chief did not try to ensure that women faculty are not subjected to subtle gender-based biases (42%), perceptions that women faculty are more likely to have others take credit for their work (42%), and perceptions that women faculty were discouraged from raising concerns about biases against women (47%). More than half reported that they felt uncomfortable raising issues about the supportiveness of the work environment for women (51%). Similarly, more than half held perceptions that reducing one’s workload hurts a woman’s chances of succeeding in her career (57%), that an obstacle for full-time women faculty is the expectation of a 60-hour work week (62%), and that women faculty who reduce their workload are viewed by their colleagues as less committed to their careers (65%).

**Table 2.** Culture Conducive to Women’s Academic Success

CCWAS scale	Mean score ± SD	Maximum possible score
Total score (4 dimensions)	143.7 ± 43.5	225
Subscale scores		
Equal access	60.4 ± 20.5	95
Gender bias	7.8 ± 3.5	15
Chair support	42.5 ± 12.6	60
Work–life balance	30.9 ± 10.4	55

CCWAS, Culture Conducive to Women’s Academic Success; SD, standard deviation.

### Burnout and fulfillment

We found that about 60% of the women in our sample reported some degree of burnout (Table 3). The PFI breaks the hypothetical construct of “burnout” down into two domains—work exhaustion and interpersonal disengagement—allowing us to parse these two contributing factors within generalized professional fulfillment. We found that the women in our sample reported feeling fulfilled at work regardless of whether they had children (Table 4). When compared with the women who did not have children, the women who had children scored higher on average in professional fulfillment and lower on average in work exhaustion and interpersonal disengagement.

**Table 3.** Burnout Screening Questions

Screening tool query	Agreed with statement *n* (%)^a^
1. I enjoy my work, I have no symptoms of burnout	2 (4.6%)
2. Occasionally I am under stress and I don’t always have as much energy as I once did, but I don’t feel burned out	15 (34.9%)
3. I am definitely burning out and have one or more symptoms of burnout, such as physical and emotional exhaustion	15 (34.9%)
4. The symptoms of burnout that I am experiencing won’t go away. I think about frustration at work a lot	9 (20.9%)
5. I feel completely burned out and often wonder if I can go on. I am at a point where I may need some changes or may need to seek some sort of help	2 (4.6%)

^a^ 43 survey respondents answered these questions.

**Table 4.** Professional Fulfillment

Professional Fulfillment Index	Mean score ± SD	Maximum possible score
Subscale scores		
Professional fulfillment	14.7 ± 4.9	24
Participants with children	15.4 ± 5.2	
Participants without children	12.5 ± 3.4	
Work exhaustion	7.8 ± 3.5	16
Participants with children	7.4 ± 3.6	
Participants without children	8.9 ± 2.9	
Interpersonal disengagement	7.4 ± 4.1	24
Participants with children	6.8 ± 4.1	
Participants without children	9 ± 3.5	

SD, standard deviation.

### Work–family conflict

Each of the women in our sample experienced some level of conflict between work and family (Table 5). The majority of the women in our sample reported feeling as though work infringed on their home and family life, but most felt that they performed well at work and did not allow family matters to interfere with work (Table 5).

**Table 5.** Work–Family Conflict

MMWFC scales	Mean score ± SD	Maximum possible score
Overall	41.2 ± 9.3	70
Time-based interference subscale	17.6 ± 4.6	30
Strain-based interference subscale	16.1 ± 4.4	30

**Individual items**	**Agreed or strongly agreed** ***n* (%)**	**Disagreed or strongly disagreed** ***n* (%)**
My work keeps me from my family activities more than I would like (*n* = 44 responses)	25 (56.8%)	
I am often so emotionally drained when I get home from work that it prevents me from contributing to my family (*n* = 45 responses)	24 (53.6%)	
Due to all the pressures at work, sometimes when I come home, I am too stressed to do the things I enjoy (*n* = 45 responses)	29 (64.4%)	
Because I am often stressed from family responsibilities, I have a hard time concentrating on my work *n* = 46 responses)		34 (73.9%)
Tension and anxiety from my family life often weakens my ability to do my job (*n* = 46 responses)		35 (76.1%)
Due to stress at home, I am often preoccupied with family matters at work (*n* = 46 responses)		30 (65.2%)

MMWFC, Multidimensional Measure of Work Family Conflict; SD, standard deviation.

### What role does childcare play?

The majority of the sample reported that availability of childcare in their area is not adequate to meet their family’s needs (70%), and that a lack of reliable childcare affects their job productivity (60%), affects the AMC’s ability to recruit women faculty (87%) and residents (80%), and influences their desire to continue working at this AMC (55%). When we examined if lack of childcare was correlated with burnout, we found that 46% of the women who reported not having family or friends available to help with childcare described feeling significantly burned out at work, whereas only 16% of the women who reported having family or friends available to help with childcare described feeling similarly burned out. Work–family conflict, as measured by the MMWFC, was increased in the women who did not have family or friends available to help with childcare when needed (43.4 ± 10.12) compared with the women who had this support (40.1 ± 8.83).

When asked about ways to improve childcare deficits in the area, qualitative responses from the sample included the need for a hospital-sponsored childcare facility, with extended hours to accommodate those working 12-hour shifts, early mornings, or late nights. Women also reported the need for drop-in care, part-time care, and facilities open on weekends and holidays. Participants suggested a network of care providers who are available for sick-care, more updated family leave policies, and partnerships with daycares that could offer longer hours or subsidized rates. Additionally, one respondent indicated that improvement could be gained by changes to the culture of human resources to be more supportive of common diversity, equity, and inclusion practices. Importantly, the women in our sample believed that when the AMC/hospital system provides these childcare benefits, it improves the climate for all faculty and especially for women.

## Discussion

The purpose of this study was to examine the aspects of workplace culture that differentially impact women at a large AMC in the Appalachian region. Our findings revealed that many women faculty felt that they were being treated differently than men faculty, that work infringed on their home and family life, and that while they were professionally fulfilled at work, childcare problems exacerbated feelings of wanting to leave that AMC.

This aligns with prior research examining the experiences of women faculty.^2,4,6^ Many in our sample reported the perception they were not as frequently considered for leadership positions as men colleagues. In other words, the women in our sample felt that men were more likely to have leadership positions in health care settings (*e.g.,* be a CEO or a department chair, *etc.*) and, as a result, might be more likely to promote other men to leadership positions. The literature is inundated with similar findings. In one such study, survey results from 614 faculty members indicated that gender significantly influenced both promotion and leadership seeking.^36^ Some of this variance could be attributable to attrition (*i.e.,* women are leaving medicine at greater rates than men), or the discrimination women faculty face when balancing parenthood with a medical career, or because of subtle gender-based biases that can occur day to day in the workplace.^36,37^

The majority of our sample reported that they perceived their chair did not take steps to ensure that subtle gender-based biases did not occur. This is in keeping with current findings, such that 60% of women physicians in emergency medicine reported having experienced gender-based discrimination.^38^ Since the civil rights movement, most forms of blatant prejudice have declined, but more covert gender-based discrimination continues to exist.^39^ Examples include being talked over, being assigned more “housekeeping” tasks, exposure to sexist humor and comments, and expectations of nurturing behavior that are not expected of men faculty.^2,40–43^ Women postgraduate surgery trainees have reported making significant physical and social adaptations to fit into their roles as surgeons, with the subsequent fatigue and burnout expected from performing the roles of a surgeon while making such significant adaptations.^39^

A major finding of our study was that the women we surveyed reported feeling burned out and as though work infringed on their home and family life. Interestingly, however, the women did not greatly endorse feeling symptoms of exhaustion or interpersonal disengagement at work. These findings suggest that, due to juggling the demands of work and family life and facing the aforementioned stressors at work, women are experiencing burnout inwardly but may be making concerted efforts to outwardly perform their job with strict professionalism. Such actions in the face of significant burnout likely serve to exacerbate feeling burned out, which may lead to women leaving the field of academic medicine early in their careers.^39^ Perhaps even more surprisingly, despite feeling burned out, our sample described feeling fulfilled at work. As far as we know, this is the first study to delineate this interesting dichotomy—work exhaustion and professional fulfillment may be independent constructs. Moreover, the women who had children tended to score higher on average in professional fulfillment than the women who did not have children; in this way, having children might have served as a protective factor by increasing professional fulfillment at work. Burnout at work in women faculty could be a function of working in a man-centric environment where they face chronic daily covert discrimination along with the multiple roles they juggle at home and at work.

Last, a vast majority of our sample described childcare options as inadequate to meet their family’s needs. Experiencing childcare problems was associated with a desire to seek employment elsewhere—a potential contributing problem to women leaving medicine. The women who participated in our survey had many ideas to help remedy this problem. Having these options available at AMCs could level the playing field for women faculty, could reduce the burnout associated with finding childcare, and perhaps decrease the number of women who leave academic medicine.

### Need for reform

The relationship between burnout, sense of professional fulfillment, and professional outcomes is complex and may be impacted by additional personal and organizational factors. In a recent study of over 18,000 physicians at AMCs, >30% reported at least moderate intent to leave their current institution in the next 2 years, and the intent to leave was most strongly associated with burnout and lack of professional fulfillment.^44^ Physician data indicated opposing effects of burnout and professional fulfillment, such that each one-point increase in burnout score was associated with 52% higher odds of intent to leave, whereas each one-point increase in professional fulfillment was associated with 36% lower odds of intent to leave.^44^ Other factors that reduced intent to leave included supportive leadership behaviors, peer support, alignment in personal and organizational values, perceived gratitude, organizational support during COVID-19, and electronic health record support, but personal factors, such as depression and negative impacts of work on personal relationships, increased intent to leave.

Similarly, Harry and colleagues found in a survey of over 58,000 health care workers that increased childcare stress was associated with increased intent to leave their current position during the COVID-19 pandemic.^30^ Of particular note, childcare stress was reported more frequently among persons identifying as having a racial or ethnic minority identity and among women. Furthermore, elevated rates of childcare stress were associated with greater odds of demonstrating anxiety, depression, or burnout.^30^ The challenges in finding childcare in rural and Appalachian communities are compounded by approximately 60% of rural communities being designated as childcare deserts and <8% of center-based childcare offering services outside of the typical workday schedule.^45^

Childcare stress is not the only factor related to work–life balance that impacts burnout and job satisfaction. Among rural medical providers, increased conflict between work and family life significantly decreases job satisfaction, increases reported burnout, and increases the likelihood of rural medical providers searching for another job.^46^ Working during personal time also predicts significantly increased stress. These findings are meaningful in the Appalachian region of the United States, where one in four counties are classified as rural and additional socioeconomic factors, such as older population, higher unemployment, lower median household income, and decreasing population relative to other rural areas of the United States may exacerbate health care disparities.^47^ This is evident in the number of designated health-care-provider-shortage areas in the Appalachian region, where 70% of nonmetropolitan areas are mental-health-professional shortage areas, and the supply of primary care physicians is 12% lower for all Appalachian counties and 26% lower in rural Appalachian counties than national averages.^48,49^

Any discussion of burnout in the professional context would be incomplete without a discussion of factors that could enhance resilience and slow or reverse the impact of burnout. Supporting faculty vitality in the academic medicine context may help accomplish this goal. Faculty vitality refers to both contextual factors and personal factors, which enhance morale and meaningful engagement in personal and professional growth.^50^ Key contextual factors may include institutional security, stability, tangible correlates of work, and shared values between the institution and the faculty. Personal factors related to faculty vitality and personal engagement include valuing excellence in clinical care, community service, teaching, and academic freedom; however, faculty often experience AMC organizational practices as impeding these values.^51^ Indeed, Dankoski and colleagues found that faculty career and life management (*e.g.*, autonomy in managing their time and balancing demands, mentorship) and positive support of faculty by institutional leadership predicted enhanced faculty vitality, measured as professional engagement, career satisfaction, and productivity.^52^

Unfortunately, institutional leadership often misidentifies the professional development and support needs of faculty, focusing on commitment to the institution or job skill development, whereas faculty prioritize support in balancing personal and professional lives, finding meaning in their work, relationships, and personal growth.^53^ Extending this literature to our current findings, interventions, such as institutional support for high-quality childcare for women faculty, expanded valuation of service in the promotion and tenure process, and enhanced formal mentorship, could substantially enrich faculty vitality for women at AMCs.

The women in our sample came up with ideas to reduce stress that focused on the need for better childcare solutions. Further ideas from the literature include more autonomy at work, greater control over working conditions, fair compensation, personal rewards, and a sense of ownership. Each of these ideas is associated with a lower rate of women who report considering leaving medicine.^37^ In addition, promotion of more women to leadership positions is necessary, not just for retaining women faculty, but because women CEOs are associated with better outcomes for the hospital, in general.^54^

Most formal studies of the impact of mentorship programs for women in AMCs focus on recruitment, promotion, and tenure for junior faculty and employ a dyadic mentor/mentee model.^55^ A recent review of this literature revealed that regardless of program design, these mentorship programs generally yield high participant satisfaction and improved rates of promotion and tenure for women faculty.^55^ However, there persists an unmet need for increased mentorship of mid-career women faculty, particularly in identifying and pursuing available career growth options, leveraging networks to achieve goals, and making career progress while balancing home responsibilities.^56^ Combining individual mentorship with intentional institutional support is likely to yield optimal outcomes, provided programs are designed with input from women faculty and needs assessments. In addition, sponsorship for specific leadership opportunities is particularly impactful for mid-career women faculty.^56^

Further, having a focus on mentoring for women faculty has proven especially helpful in career advancement and decreasing the pay gap. A curriculum called the Career Development Programs (CDPs) has shown efficacy in improving both promotion and increasing salaries for women physicians.^57^ Implementing formal mentoring systems for new women faculty by late-career women faculty who are paid for this work or through internet-based curriculums, such as the CDP, is a prudent action for all AMCs.^57^ As noted, there are many avenues for changes that may decrease the stress and burnout faced by women physicians. If a focus is given to decreasing that stress, we may see a greater retention of women faculty, which in turn will mean more women in the highest positions in health care and academia in the years to come.

### Strengths and limitations

Strengths of this study lie in the multiple avenues for which it has incrementally increased validity. First, women’s experiences in academic medicine in the heart of Appalachia have been understudied. Similar studies include samples from the Midwest or the Southern regions of the United States, but Appalachia has unique cultural aspects, so this study adds new information to the existing literature. Our finding that the women at our AMC might be burned out but are still fulfilled at work might be attributed to factors unique to the Appalachian region. We also provide incremental validity by replicating the findings of previous studies, namely that childcare is a major stressor for women faculty, that women feel burned out in health care, and that women face work–family conflicts.^22,26,28^ Given the recently identified “replication crisis” in academic psychology,^58^ any areas where we can provide replication of results are important and necessary. Finally, this study provides incremental validity to the use of several measures that do not currently have a vast amount of literature supporting their psychometric properties. These measures have demonstrated validity and reliability in certain populations, and our findings add to this by providing data from a sample of women working in health care in Appalachia.

The greatest limitation of this study lies in the small sample size, all of whom self-selected into the study. The response rate to the survey was low. The sample composition was mostly non-Hispanic, white, and was completely cisgender; thus, there was underrepresentation of women faculty in minority groups in the study. Compared with data from the AAMC, the participants in this study may underrepresent academic rank progression and leadership roles compared with other AMCs.^35^ Because of the small sample, we were limited in statistical power. Having limited statistical power meant that we could not perform inferential analyses as we would have liked, and some findings should be interpreted with caution until further replication can be performed. Furthermore, because our sample was limited to women, we could not draw comparisons between men and women on constructs such as burnout.

## Conclusions

In sum, this study explored the experiences of women faculty at a large AMC in the Appalachian region of the United States in a sample of 47 women practicing in different areas of medicine. We found that the women felt that they were treated differently than men faculty, were burned out but felt fulfilled at work, and reported that difficulty with childcare availability in this region adds to the desire to leave the area. Due to desires for equality and to prevent the subsequent health and mental health problems that arise from stress, we include ideas to remedy some of these problems, including on-site childcare facilities and mentorship for women faculty, among other ideas. Taken together, this article demonstrates that there is great room for improvement in eliminating the differential treatment between men and women faculty in academia. We conclude by echoing the words of an editorial in the *New England Journal of Medicine* published in 1849 that “no law prevents women from occupying…the three great fields of medicine…but there are obstacles nevertheless, much more subtle and powerful than law.”^59^

## Data Access Statement

Electronic study data are stored on a secure research drive at the CAMC Institute of Academic Medicine. The authors will honor reasonable requests from outside parties to access data.

## Abbreviations Used

AAMC: American Association of Medical Colleges
AMC: Academic Medical Center
CCWAS: Culture Conducive to Women's Academic Success
CDP: career development programs
IRB: Institutional Review Board
MMWFC: Multidimensional Measure of Work Family Conflict
PFI: Professional Fulfillment Index
